# Gravitropism and Lateral Root Emergence are Dependent on the *Trans*-Golgi Network Protein TNO1

**DOI:** 10.3389/fpls.2015.00969

**Published:** 2015-11-12

**Authors:** Rahul Roy, Diane C. Bassham

**Affiliations:** ^1^Department of Genetics, Development and Cell Biology, Iowa State University, AmesIA, USA; ^2^Interdepartmental Genetics Program, Iowa State University, AmesIA, USA; ^3^Plant Sciences Institute, Iowa State University, AmesIA, USA

**Keywords:** auxin, *trans*-Golgi network, lateral root, gravitropism, tethering factor

## Abstract

The *trans*-Golgi network (TGN) is a dynamic organelle that functions as a relay station for receiving endocytosed cargo, directing secretory cargo, and trafficking to the vacuole. TGN-localized SYP41-interacting protein (TNO1) is a large, TGN-localized, coiled-coil protein that associates with the membrane fusion protein SYP41, a target SNARE, and is required for efficient protein trafficking to the vacuole. Here, we show that a *tno1* mutant has auxin transport-related defects. Mutant roots have delayed lateral root emergence, decreased gravitropic bending of plant organs and increased sensitivity to the auxin analog 2,4-dichlorophenoxyacetic acid and the natural auxin 3-indoleacetic acid. Auxin asymmetry at the tips of elongating stage II lateral roots was reduced in the *tno1* mutant, suggesting a role for TNO1 in cellular auxin transport during lateral root emergence. During gravistimulation, *tno1* roots exhibited delayed auxin transport from the columella to the basal epidermal cells. Endocytosis to the TGN was unaffected in the mutant, indicating that bulk endocytic defects are not responsible for the observed phenotypes. Together these studies demonstrate a role for TNO1 in mediating auxin responses during root development and gravistimulation, potentially through trafficking of auxin transport proteins.

## Introduction

The *trans*-Golgi network (TGN) is a highly dynamic tubulo-vesicular organelle that matures from the two or three *trans*-most cisternae of the Golgi (Staehelin and Kang, 2008) and is crucial for endocytic, secretory and vacuolar trafficking routes in plant cells. TGN cisternae move rapidly inside the cell, dissociating from their associated Golgi and re-associating with a new Golgi stack (Staehelin and Kang, 2008; Kang, 2011; Uemura et al., 2014), and also contain distinct subdomains for various trafficking routes (Bassham et al., 2000; Chow et al., 2008; Gendre et al., 2011). The TGN functions as an early/recycling endosome (Dettmer et al., 2006; Viotti et al., 2010) that receives endocytosed cargo, including auxin transporters, plasma membrane receptors, and nutrient transporters (Russinova et al., 2004; Robatzek et al., 2006; Dhonukshe et al., 2007; Takano et al., 2010; Barberon et al., 2011), and recycles it back to the plasma membrane or to the vacuole for degradation. The TGN plays a crucial role in trafficking of biosynthetic traffic to the vacuole (Reyes et al., 2011). It also directs secretory cargo, including plasma membrane proteins and cell wall polysaccharides, to the cell surface, potentially via mobile secretory vesicle clusters that fuse with the plasma membrane (Toyooka et al., 2009; Gendre et al., 2014). The position of the TGN at the junction of the endocytic, vacuolar, and secretory pathways renders it important in regulating transport of key molecules and mediating cellular responses to the environment (Park and Jurgens, 2011; Reyes et al., 2011; Contento and Bassham, 2012).

High transport fidelity is needed to prevent mis-sorting of cargo during vesicle trafficking. This requires membrane fusion proteins termed soluble *N*-ethylmaleimide-sensitive factor attachment protein receptors (SNAREs; Risselada and Grubmuller, 2012). SNAREs can be broadly classified as target SNARES (t-SNAREs) or vesicle SNAREs (v-SNAREs) depending on their location, or as Q or R-SNAREs based on the core amino acid in the heptad repeat of the SNARE motif (Fasshauer et al., 1998). The interaction between a v-SNARE on a vesicle and t-SNAREs on its target membrane leads to membrane fusion via formation of a tetrameric *trans-*SNARE complex (McNew et al., 2000; Lipka et al., 2007; Kim and Brandizzi, 2012; Risselada and Grubmuller, 2012). Usually, three Q SNAREs (Qa, Qb, and Qc) form a t-SNARE complex and an R-SNARE acts as the v-SNARE. This interaction of SNAREs helps to overcome the thermodynamically unfavorable event of fusion of two hydrophobic lipid bilayers (Risselada and Grubmuller, 2012; Shi et al., 2012), enabling deposition of cargo into the target organelle. Proteins known as tethering factors aid in bringing membranes together and promoting SNARE interaction or actively stimulating *trans*-SNARE complex formation (Cai et al., 2007). Tethering factors thus increase vesicular trafficking efficiency (Sztul and Lupashin, 2009; Chia and Gleeson, 2011; Hong and Lev, 2014) and are either homodimeric long coiled-coil proteins or multisubunit tethering complexes (Markgraf et al., 2007; Hong and Lev, 2014). It was recently proposed that coiled-coil tethers simultaneously use their multiple coiled-coil domains to engage distinct SNAREs and promote SNARE complex assembly (Grabski et al., 2012). Spatiotemporal regulation of trafficking steps in the cell also requires a family of small GTPases called Rabs which then recruit downstream effectors (Stenmark, 2009). Tethers can act as Rab effectors by binding SNAREs or can function as GTP exchange factors (GEFs) for Rabs (Markgraf et al., 2007; Sztul and Lupashin, 2009; Hong and Lev, 2014).

Many SNAREs and tethering factors exist in *Arabidopsis thaliana*, with different localizations reflecting their specialized roles (Fujimoto and Ueda, 2012). The TGN-localized SYP4 (41/42/43) SNARE family (Bassham et al., 2000; Uemura et al., 2012) is responsible for maintaining Golgi/TGN morphology and regulating secretory and vacuolar trafficking (Uemura et al., 2012). Another TGN SNARE, SYP61, interacts with SYP41 and helps to direct traffic to the plasma membrane (Drakakaki et al., 2012), with a role in mediating stress responses (Zhu et al., 2002). Recently, TGN-localized SYP41-interacting protein (TNO1), a large coiled-coil protein localized to the TGN, was identified as a SYP41 interactor (Kim and Bassham, 2011) and was hypothesized to be a tethering factor. Mutant plants lacking TNO1 partially mis-sort vacuolar cargo and mis-localize SYP61, suggesting decreased trafficking fidelity, while also showing hypersensitivity to salt and osmotic stress and displaying altered TGN dynamics (Kim and Bassham, 2011).

SYP42 and SYP43 play a role in root gravitropism, most likely via regulation of the localization of the auxin eﬄux transporters PIN-FORMED 1 (PIN1) and PIN2 (Uemura et al., 2012). Additionally, other proteins involved in auxin transport, such as the AUXIN1/LIKE-AUX1 (AUX1/LAX) family of auxin influx transporters and P-glycoprotein (PGP) proteins of the ATP-binding cassette transporter family, are localized via the SNAREs and TGN activity (Kleine-Vehn and Friml, 2008; Rakusová et al., 2015). Many of the auxin transporters undergo constitutive endocytosis, cycling between the recycling endosome/TGN and the plasma membrane, or are targeted for vacuolar degradation to maintain steady-state levels (Kleine-Vehn and Friml, 2008; Grunewald and Friml, 2010). Defects in TGN dynamics can, therefore, hamper recycling of these transporters and thus affect directional transport of auxin, which is critical for plant development (Grunewald and Friml, 2010).

Given the potential links between TGN-mediated protein trafficking and auxin transporters, we investigated auxin responses in the *tno1* mutant during root development. Loss of TNO1 delayed lateral root (LR) emergence and decreased root and hypocotyl gravitropic bending. Additionally, *tno1* roots failed to display characteristic asymmetry visualized with the auxin response marker *DR5rev:GFP* at the LR tips as well as after gravistimulation. Thus, TNO1 functions in auxin-mediated root development and response to gravity.

## Materials and Methods

### Plant Material and Growth Conditions

The *A. thaliana* seed stocks used in this study have been previously described: Col-0 (wild-type, WT), *tno1* knockout mutant (SALK_112503; Kim and Bassham, 2011), complemented *tno1* mutant (Kim and Bassham, 2011), and *DR5rev:GFP* (Ottenschlager et al., 2003).

*Arabidopsis* seeds were surface-sterilized in 33% bleach, 0.1% (v/v) Triton X-100 for 20 min, rinsed five times with sterile water and kept in the dark at 4°C for at least 2 days. *Arabidopsis* plants were grown at 22°C in long-day conditions (16 h light) in soil or on 0.25× or 0.5× solid Murashige–Skoog (MS) medium (MS vitamin and salt mixture, Caisson, MSPA0910) with 1% sucrose, 2.4 mM MES (pH 5.7), and 0.6% (w/v) phytoblend agar (Caisson, PTP01).

### Gravitropism Assays

The hypocotyl gravitropism assay was modified from Stanga et al. (2009). Seeds were plated on 0.5× MS medium (Murashige and Skoog, 1962) containing 1% sucrose in square plates and kept vertically oriented in the dark. After 5 days, the plates were rotated 90° for gravistimulation. Pictures were acquired using a Canon Rebel XTS camera in a dark room with a green filter over the flash to prevent phototropic curvature of the hypocotyl toward the camera flash. Zero-hour images of each seedling were compared to later time point images of the same seedling using Image J (Schneider et al., 2012) to determine bending angles.

For root gravitropic assays, sterilized seeds were mixed with molten growth medium just before solidification and poured into square plates (Stanga et al., 2009). After 5 days of vertical growth the seedlings were gravistimulated by rotating the plate by 90°. Pictures were taken 6 and 24 h after gravistimulation and analyzed using Image J to assess gravitropic curvature.

For root and hypocotyl gravitropic rescue assays, the roots of 5-day-old vertically grown seedlings were overlaid with media containing either 100 nM 1-napthylacetic acid (1-NAA; Sigma–Aldrich, N0640), 30 nM 3-indoleacetic acid (IAA; Sigma–Aldrich, I2886), or 30 nM 2,4-dichlorophenoxyacetic acid (2,4-D; Gibco, 11215), followed by gravistimulation. Pictures of roots and hypocotyls after 12 h were compared to 0-h images using Image J. For all assays, at least three biological replicates were performed, with 15–20 seedlings per replicate.

### LR Density Analysis

To determine the density of emerging LRs, sterilized *Arabidopsis* seeds were plated on 0.25× MS medium (Murashige and Skoog, 1962) containing 1% sucrose and grown vertically. After 10 days, the number of LRs emerging from the primary root were counted and divided by the root length to obtain LR density. For determination of LR primordia density, 7-day-old roots were cleared with 2.5% bleach for 10 min and visualized with an Olympus IX-71 inverted microscope. To assess rescue of LR emergence, 5-day-old seedlings were transferred to medium containing 100 nM 1-NAA or 1 μM IAA in the dark (to prevent photo-degradation). After 5 additional days, the number of emergent LRs was scored. For each analysis, three independent biological replicates were performed with 15–20 seedlings per replicate.

### Root Length Inhibition Assays

Seedlings were grown on 0.5× MS medium (Murashige and Skoog, 1962) with 1% sucrose for 5 days. They were then transferred to media containing either 2,4-D, IAA, 1-NAA, 1-*N*-naphthylphthalamic acid (NPA; Naptalam, Sigma–Aldrich, 33371), 1-napthoxyacetic acid (1-NOA; Sigma–Aldrich, 255416) at the concentrations indicated, or solvent controls [dimethyl sulfoxide (DMSO) or ethanol], and the position of the root tip was marked. Each plate contained all three genotypes (WT, *tno1* mutant, and complemented lines) to compensate for possible effects of inter-plate variation. After 6 days, pictures were acquired and the length from the marked root tip position was measured using ImageJ (Schneider et al., 2012). Root elongation in the presence of the tested chemicals was compared with solvent controls and expressed as percent decrease in root length. At least three biological replicates with a minimum of 20 seedlings per replicate were conducted for each treatment.

### Analysis of Auxin Response Distribution

*tno1* plants expressing *DR5rev:GFP* were generated by crossing *tno1* (Kim and Bassham, 2011) with *DR5rev:GFP* lines (Ottenschlager et al., 2003). Homozygous *tno1* plants carrying the *DR5rev:GFP* transgene were selected in subsequent generations by polymerase chain reaction (PCR)-based genotyping as previously described (Kim and Bassham, 2011) and green fluorescent protein (GFP) fluorescence visualization. For analysis of auxin redistribution, control and *tno1* roots expressing *DR5rev:GFP* were gravistimulated and visualized with a Leica SP5 confocal laser scanning microscope (Leica Microsystems) at the Iowa State University Confocal and Multiphoton facility. A 40× oil immersion objective lens was used along with excitation and emission wavelengths of 488 and 507 nm for GFP visualization. Images were acquired under identical conditions for both mutant and WT roots with equal exposure, scan frequency and line average settings. A total of 15 seedlings from at least three independent replicates were analyzed. GFP asymmetry was quantified using Image J by subjecting the confocal images to similar thresholding and post-processing. Equal volume boxes were drawn on the upper and lower flanks and total pixel intensity from the lower vs. upper box was expressed as a ratio. To analyze *DR5rev:GFP* expression in LRs, a total of 30 stage II LRs from at least six different seedlings were imaged by confocal microscopy for each genotype. The number of root tips showing asymmetry of GFP expression was counted and expressed as a percentage of the total observed.

### FM4-64 Staining and Brefeldin A (BFA) Treatment

FM4-64 staining was modified from Dettmer et al. (2006). To test bulk endocytosis, 4-day-old seedlings were transferred to 0.5× MS liquid medium containing 4 μM FM4-64 for 2 min and subsequently washed twice for 30 s each time in 0.5× MS liquid medium before microscopic analysis. For analyzing arrival of FM4-64 at Brefeldin A bodies, 4-day-old seedlings were transferred to 0.5× MS liquid medium containing 35 μM Brefeldin A (BFA) for an hour followed by a 10-min treatment with 4 μM FM4-64 plus 35 μM BFA and two subsequent washes of 30 s each. The root tips were visualized using a Leica SP5 confocal laser scanning microscope (Leica Microsystems) at the Iowa State University Confocal and Multiphoton facility, using a 63× oil immersion objective lens and excitation and emission wavelengths of 558 and 734 nm. Images were acquired under identical conditions for both genotypes with equal exposure, scan frequency and line average settings. A total of 15 seedlings from at least three independent replicates were analyzed for each treatment and genotype.

## Results

### TNO1 Mediates LR Emergence in an Auxin-dependent Manner

**FIGURE 1:**
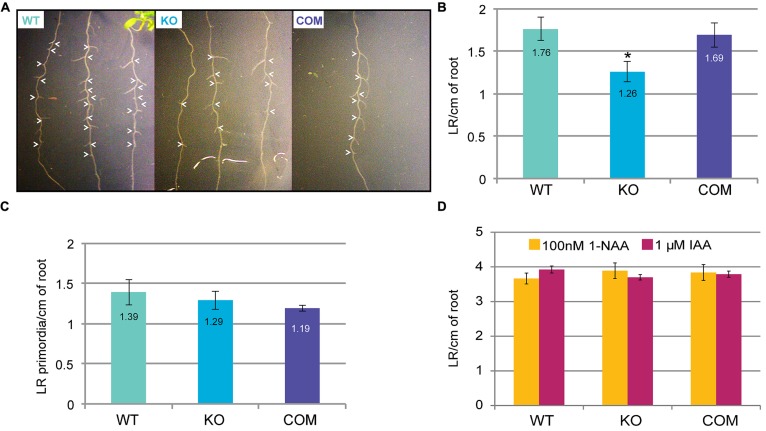
**Loss of TGN-localized SYP41-interacting protein **(**TNO1) function delays lateral root emergence in an auxin-dependent fashion.** **(A)** Representative images showing emergent lateral roots (white arrowheads) in wild-type (WT), *tno1* (KO), and *tno1* complemented (COM) lines. **(B)** Emergent lateral root density of 10-day-old seedlings grown on 0.25× Murashige–Skoog (MS) medium (with 1% sucrose) was calculated by dividing the number of emerging lateral roots by root length. **(C)** Lateral root primordia density of 7-day-old seedlings was calculated by counting the number of primordia after microscopic analysis of cleared roots and dividing it by the root length. **(D)** Emergent LR density of 10-day-old seedlings, 5 days after transfer to medium containing 100 nM 1-napthylacetic acid (NAA) or 1 μM indoleacetic acid (IAA). All values represent analysis of three biological replicates with 15–20 seedlings for each set. Error bars indicate standard errors of the means. Asterisk indicates statistically significant difference (*P* < 0.05) by Student’s *t*-test.

Auxin signaling pathways are integral to LR development during root architecture establishment (Lavenus et al., 2013). Polar auxin transport mediates emergence of LRs from the primary axis, and several auxin transport mutants display slow rates of emergence (Hobbie and Estelle, 1995; Ruegger et al., 1997). The SYP4 family of SNAREs is thought to be involved in directional auxin transport (Uemura et al., 2012), hence we tested whether the SYP41-interacting protein TNO1 may also be required for such processes. We hypothesized that if the *tno1* mutant has defects in auxin transport or responses, visible phenotypes such as changes in LR emergence would be evident. Emergent LR densities were significantly lower (*P* < 0.05) in *tno1* seedlings after 10 days of growth on 0.25× MS medium (Dubrovsky and Forde, 2012). Mutant lines expressing transgenic *TNO1* under the control of its native promoter, termed complemented lines (Kim and Bassham, 2011), were similar to WT seedlings (**Figures 1A,B**). Since the major source of auxin in the root in the first 10 days after germination is transport from the leaves (Hobbie and Estelle, 1995; Ljung et al., 2001), one possible reason for this defect may be that auxin flux is reduced in *tno1*, leading to suboptimal auxin levels in the root and impairing LR emergence. To confirm that the delayed emergence was not due to an arrest or delay in LR initiation, the number of LR primordia was evaluated in the mutant roots. The LR primordia density of *tno1* roots was equivalent to that of WT and complemented lines (**Figure 1C**), suggesting that LR initiation events were normal in *tno1*.

The lipophilic auxin 1-NAA has been shown to rescue LR defects in the auxin transport mutant *aux1* (Marchant et al., 2002). Natural auxin (IAA) treatment also rescues LR emergence in dark conditions (Reed et al., 1998). To test whether the emergence defect may be due to defects in auxin transport and hence reduced root auxin levels, LR emergence in the presence of 1-NAA (Murashige and Skoog, 1962; Marchant et al., 2002) or IAA in the dark (Reed et al., 1998) was assessed. Five-day-old seedlings were transferred to medium containing 100 nM NAA or 1 μM IAA, and LR density was analyzed after 5 days of growth. The LR density of *tno1* resembled that of WT and complemented lines in the presence of either auxin (**Figure 1D**), indicating that exogenous auxins can rescue the emergent LR defect in *tno1* roots. Therefore, TNO1 influences the temporal control of LR emergence from the primary root.

### TNO1 is Required for Gravitropic Bending

We further examined a possible role for TNO1 in plant auxin responses by investigating gravitropism. Gravitropism involves the bending of a plant organ in response to its change in orientation with respect to gravity (gravistimulation). Gravitropic bending of organs upon gravistimulation is aided by a readjustment of auxin flow. Since a number of proteins involved in gravitropic curvature are recycled and trafficked via the TGN (Strohm et al., 2012), this organelle is critical for regulating downstream events that influence gravitropic plant organ bending. We, therefore, hypothesized that loss of TNO1 might affect dynamics at the TGN and thus lead to a change in the gravitropic bending response.

**FIGURE 2:**
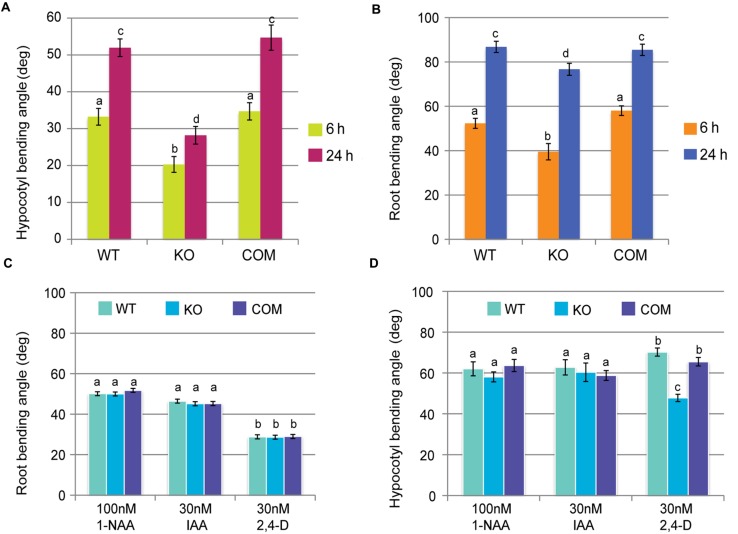
**TNO1 mediates gravitropic bending by an auxin dependent mechanism.** **(A)** Hypocotyl bending of WT, KO and COM hypocotyls after gravistimulation. Dark-grown 5-day-old hypocotyls were gravistimulated in the dark and curvatures were calculated at 6 and 24 h after gravistimulation. **(B)** Root bending of WT, KO, and COM seedlings after gravistimulation. Light-grown, medium-embedded roots were gravistimulated and root curvatures were calculated at 6 and 24 h after gravistimulation. **(C)** Exogenous auxins can rescue the gravitropic bending defect in *tno1* roots. Light-grown seedlings were overlaid with medium containing 100 mM 1-NAA, 30 nM IAA, or 30 nM 2,4-dichlorophenoxyacetic acid (2,4-D) and then gravistimulated. Root curvatures were calculated after 12 h. **(D)** Some exogenous auxins can rescue the gravitropic bending defect in *tno1* hypocotyls. Roots of dark-grown seedlings were overlaid with medium containing 100 mM 1-NAA, 30 nM IAA, or 30 nM 2,4-D, followed by gravistimulation. Hypocotyl curvatures were calculated after 12 h. All values represent the means of three biological replicates with at least 20 seedlings for each set. Error bars indicate standard errors. Similar letters indicate no statistical difference while different letters indicate a statistically significant difference (*P* < 0.05) by Student’s *t*-test.

To test this hypothesis, the gravitropic response of WT and *tno1* mutant hypocotyls and roots was assessed. The *tno1* mutant hypocotyls (**Figure 2A**) and roots (**Figure 2B**) showed delayed bending, and the angle of curvature at different time points was significantly lower (*P* < 0.05) than that of WT or complemented seedlings. This suggests that the loss of TNO1 causes a delay in the gravitropic bending of *Arabidopsis* roots and hypocotyls. Mutant roots and hypocotyls have a similar length to WT under normal growth conditions (Kim and Bassham, 2011), suggesting that the defect in gravitropism is not due to defective growth.

The gravitropic bending response requires a reprogramming of auxin flow inside the shoot and root (Tanaka et al., 2006; Strohm et al., 2012), raising the possibility that the slower bending rate observed in *tno1* could be due to changes in auxin flow. We hypothesized that if the gravitropic phenotype in *tno1* mutants is due to defect(s) in auxin transport, it may be rescued by exogenous auxin application. To test this idea, we used the membrane-permeable auxin 1-NAA, which has been previously used to rescue gravitropic root bending defects in mutants defective in auxin transport (Marchant et al., 1999), the natural auxin IAA, and the influx-specific auxin 2,4-D. Following auxin treatments, the bending angles of *tno1* roots were not significantly different (*P* > 0.1) from the bending angles of the WT and complemented lines (**Figure 2C**), indicating rescue of the gravitropic bending defect by exogenous auxin. The 2,4-D treatment led to an overall reduction of bending angles as has been previously reported (Surpin et al., 2005) but there were no significant differences between WT and mutant. Similarly, hypocotyl bending was also rescued after a 12-h treatment with either IAA or 1-NAA (**Figure 2D**), confirming that the gravitropic defect in *tno1* mutants is most likely due to auxin-related defects. 2,4-D did not rescue the gravitropic defect in the mutant hypocotyls, with bending angles significantly lower than WT and complemented lines; this is consistent with previous reports demonstrating an effect of 2,4-D on gravitropism in roots but not in hypocotyls (Surpin et al., 2005). The *tno1* mutant defects in gravitropic bending, therefore, may be due to defects in auxin transport or response.

### *tno1* Roots Show Increased Sensitivity to 2,4-D and IAA

**FIGURE 3:**
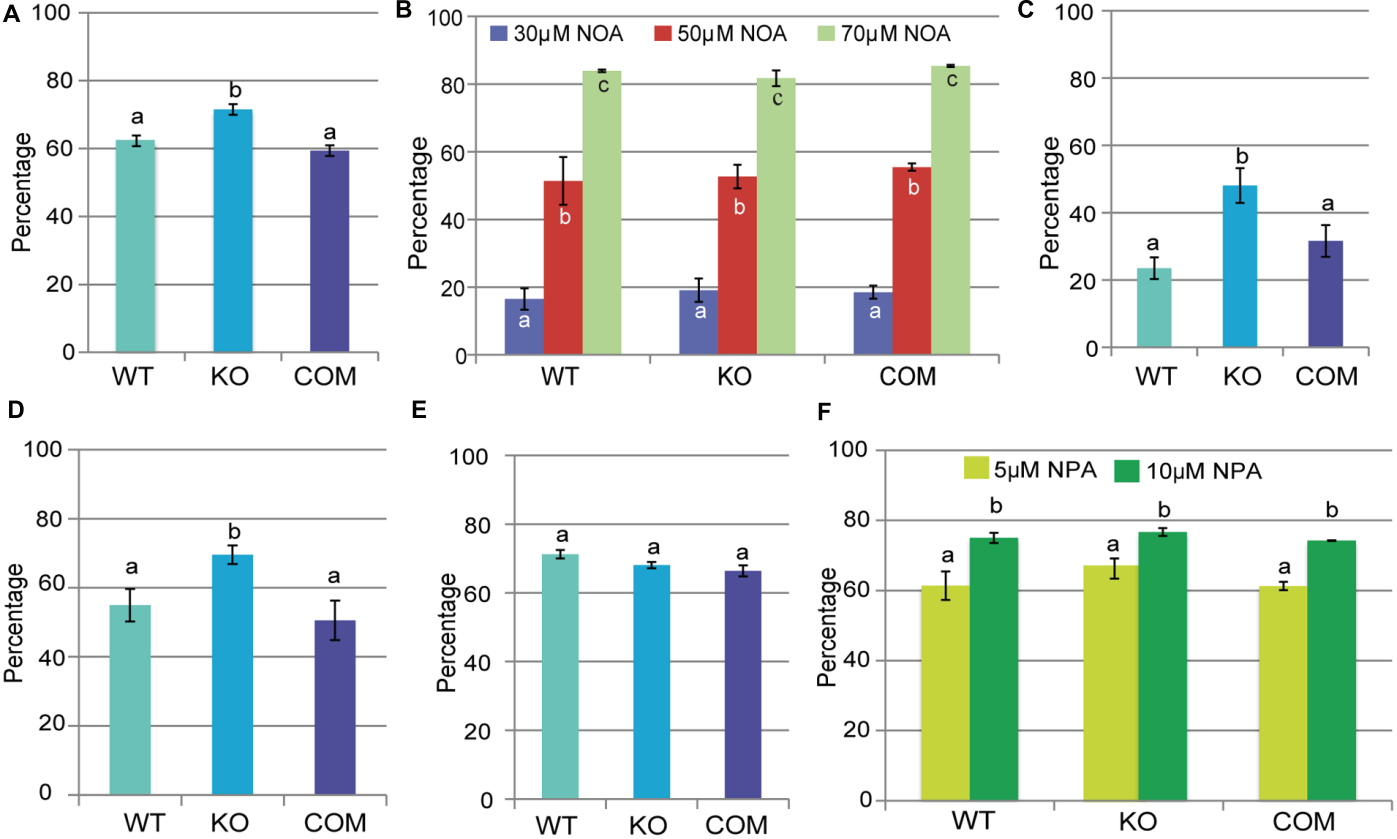
***tno1* roots show increased sensitivity to 2,4-D and IAA.** Five-day-old seedlings were transferred to media with the indicated chemical or solvent as a control and root length was calculated after an additional 6 days of growth. Mean root growth of each genotype on the chemical treatment compared to the mean on the solvent control was expressed as percentage inhibition. Percent inhibition of root length on **(A)** 30 nM 2,4-D, **(B)** 1-1-napthoxyacetic acid (NOA; 30, 50, and 70 μM), **(C)** 30 nM 2,4-D + 10 μM 1-NOA, **(D)** 40 nM IAA, **(E)** 100 nM 1-NAA, and **(F)** 1-*N*-naphthylphthalamic acid (NPA; 5 and 10 μM) for WT, KO, and COM lines are shown. Values represent analysis of three biological replicates with 20 seedlings for each set. Error bars indicate standard errors derived from means of the three replicates. Different letters indicate statistically significant differences (*P* < 0.05) by a Student’s *t*-test.

Auxin flux into plant cells is facilitated by influx carriers such as AUX1 and the LAX family of transporters, while exit from the cell involves eﬄux carriers belonging to the PIN and PGP families (Peer et al., 2011). Auxin analogs and transport inhibitors are important tools to uncover defects in auxin transport pathways. The auxin analog 2,4-D is an influx-specific substrate while 1-NAA is an eﬄux-specific substrate (Delbarre et al., 1996). The auxin transport inhibitor NPA interferes with auxin eﬄux while 1-NOA interferes with auxin influx and inhibits AUX1 (Thomson et al., 1973; Sussman and Goldsmith, 1981; Yang et al., 2006). To determine the effect of these inhibitors on the *tno1* mutant, their effects on root growth were assessed. We found that *tno1* roots were significantly (*P* < 0.05) more sensitive to 30 nM 2,4-D than WT or complemented roots (**Figure 3A**). This result is consistent with our observation of the failure of 2,4-D to rescue the gravitropic defect of mutant hypocotyls.

One possible explanation for this sensitivity is that influx routes are altered in the *tno1* mutant, which led us to test the effect of blocking auxin influx with 1-NOA. Mutant roots showed similar sensitivity to WT and complemented lines across a range of concentrations of 1-NOA (**Figure 3B**), suggesting that blocking auxin influx affects the *tno1* mutant to the same extent as wild-type. 1-NOA has a characteristic protective effect against root growth inhibition by 2,4-D (Parry et al., 2001); we, therefore, tested whether 1-NOA could rescue the root growth inhibition of mutant roots by 2,4-D to comparable levels as in the WT and complemented lines. Seedlings were transferred to medium containing 30 nM 2,4-D and 10 μM 1-NOA. The WT and complemented lines showed a significantly lower inhibition of root length than the mutants (*P* < 0.05), indicating that when auxin influx is blocked, *tno1* mutants still have enhanced sensitivity to 2,4-D (**Figure 3C**).

To determine whether the sensitivity to 2,4-D is also seen with other auxins, root growth inhibition on 40 nM IAA in the dark was assessed. *tno1* mutant roots are indeed more sensitive to IAA (*P* < 0.05) than WT or complemented lines (**Figure 3D**). By contrast, treatment with 0.1 μM of the membrane-permeable NAA inhibited the growth of *tno1* to a similar extent as WT and complemented plants (**Figure 3E**). Since 2,4-D and IAA are influx-specific substrates and NAA is not, the results are consistent with a possible defect in auxin influx pathways in *tno1*. *tno1*, WT, and complemented plants had similar responses to NPA treatment (**Figure 3F**), suggesting that the auxin eﬄux pathway is unaffected in *tno1*.

### TNO1 Helps Mediate Auxin Responses during LR Elongation

Lateral roots emerge perpendicular to the primary root and gradually start bending rootwards. Recently emerged LRs, which lack an elongation zone (stage I LR) gradually transition into stage II LRs, defined by an elongation zone and asymmetric growth of the upper and lower epidermal cell files (Rosquete et al., 2013). The differential growth peaks in the stage II LRs are due to asymmetric auxin distribution, which in turn is dependent on auxin transport mechanisms. Stage II LRs are thus defined by a characteristic asymmetric distribution of auxin response, which results in differential cellular elongation and downward root growth. This asymmetric auxin flux at the LR tip is caused by the specific activity and distribution of auxin transporters (Rosquete et al., 2013). The asymmetric auxin distribution pattern diminishes significantly as the LRs mature to Stage III and begin growing parallel to the primary root. It was reported that on average more than 75% of stage II LRs in WT plants display asymmetric distribution of auxin responses at LR tips (Rosquete et al., 2013), visualized by the pattern of *DR5rev:GFP* expression (Friml et al., 2003).

**FIGURE 4:**
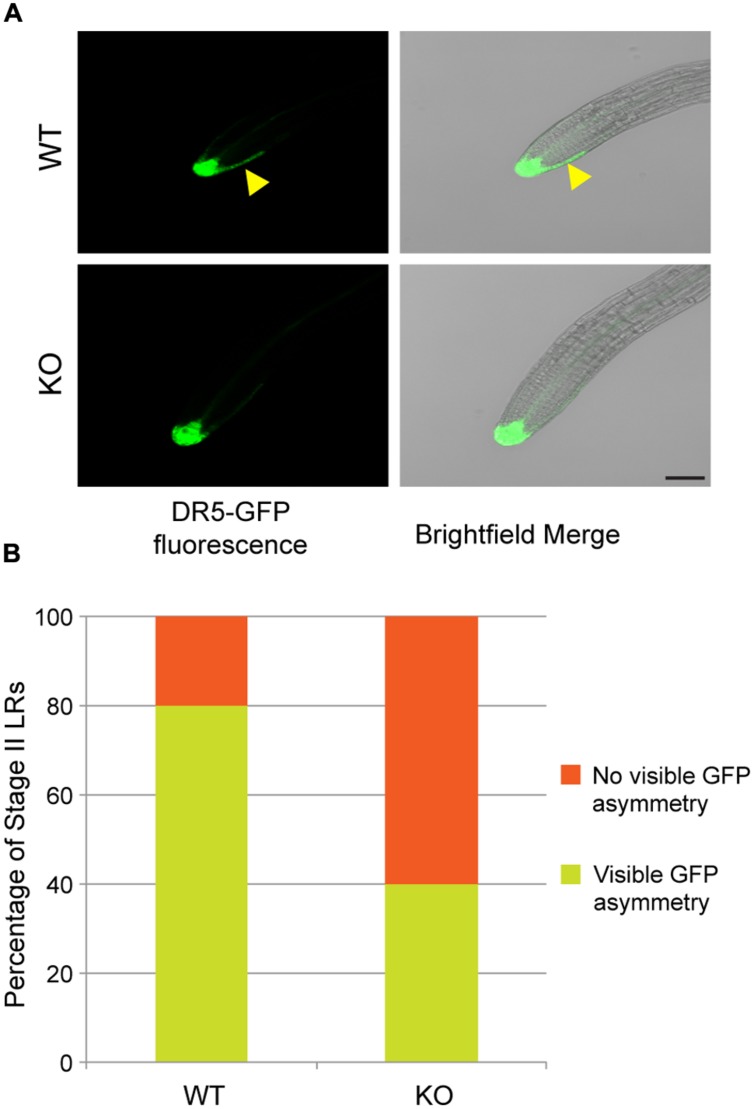
**TNO1 helps mediate auxin response asymmetry at lateral root (LR) tips.** **(A)** Representative confocal images of stage II LR tips of WT and *tno1* expressing the auxin-responsive *DR5rev:GFP* reporter. Yellow arrowheads indicate asymmetry of the auxin reporter GFP expression. Scale bar = 50 μm. **(B)** The percentage of stage II LRs displaying an asymmetric auxin response pattern. A total of 30 stage II LRs from at least six seedlings were analyzed.

Since TNO1 is linked to LR emergence by a possible auxin-dependent mechanism, we hypothesized that asymmetric auxin transport or response is defective in *tno1* LRs. Hence, the percentage of LRs showing asymmetric auxin responses in stage II *tno1* LR tips would be lower compared to WT (or complemented) root tips. To test this, the percentage of stage II LRs with auxin response asymmetry in WT and *tno1* background lines expressing *DR5rev:GFP* was determined by confocal microscopy. 40% of *tno1* stage II LRs displayed auxin asymmetry at the LR tip compared to 80% in WT stage II LRs (**Figures 4A,B**). Thus, *tno1* roots are defective in establishing the correct pattern of auxin response during LR development. This defect could explain the lower emergent LR density observed in *tno1* compared to WT, since a disruption of auxin flow or establishment of asymmetry would prevent LR emergence.

### TNO1 Facilitates Shootward Auxin Response during Root Bending

Normally, gravitropic bending of the primary root leads to an eventual return of the root tip toward the gravity vector. This occurs via a cascade of signals leading to asymmetric auxin flow from the columella cells to the lateral rootcap cells and eventually shootward through the epidermal and cortical cells (moving toward the root–shoot junction). During this process, auxin accumulates on the lower side of the root, leading to growth inhibition, while the top of the root continues to elongate, thus resulting in downward root bending (Ottenschlager et al., 2003; Kleine-Vehn et al., 2010; Baldwin et al., 2012). Since *tno1* roots are defective in gravitropic bending (**Figure 2**) and the frequency of LR tips displaying auxin response asymmetry is lower than in WT LRs (**Figure 4**), we hypothesized that the defect in root bending could be due to a decrease in shootward transport of auxin from the columella to the lateral rootcap cells and on to the distal elongation zone.

**FIGURE 5:**
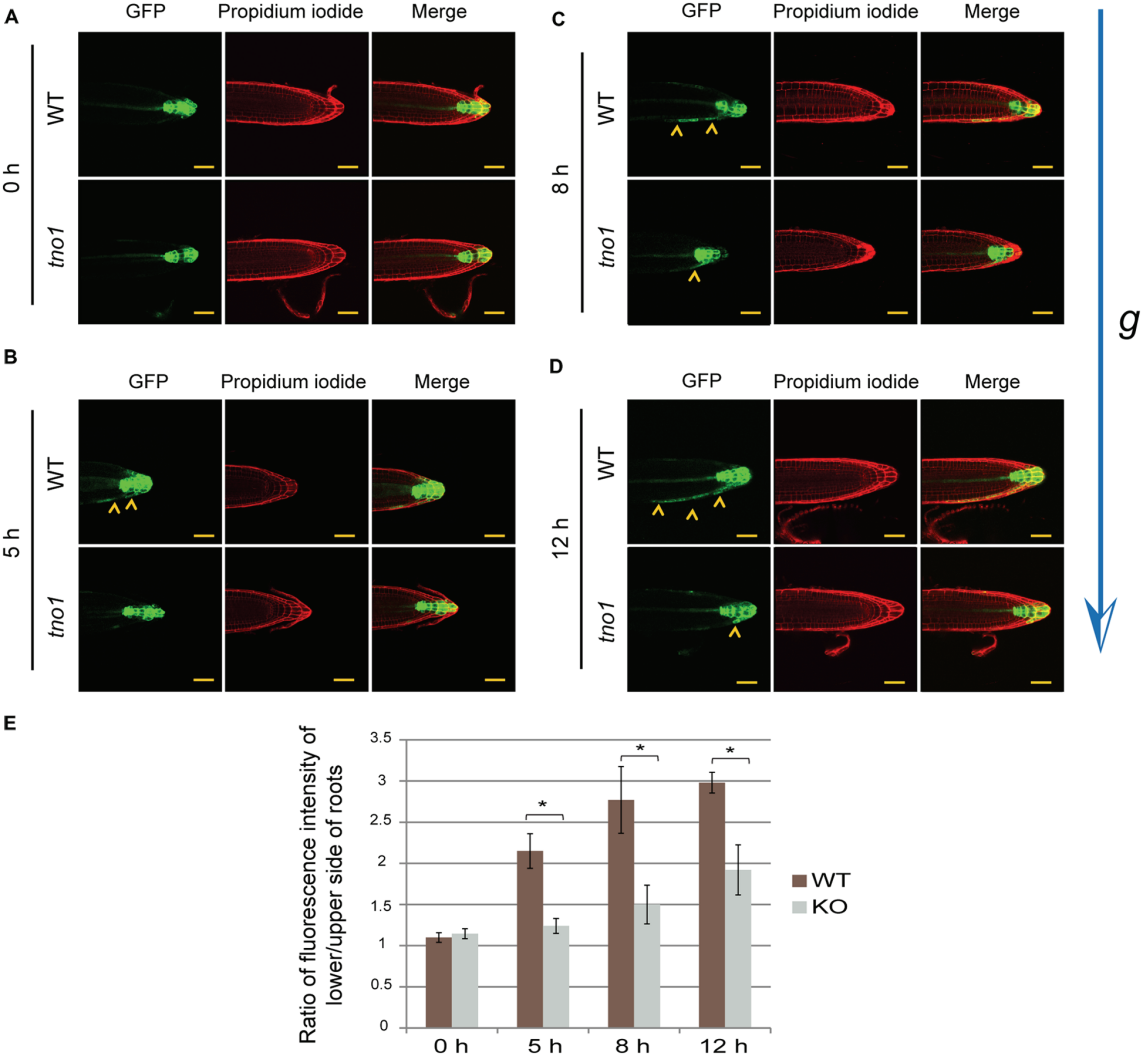
***tno1* mutants have delayed auxin responses in epidermal cells in gravistimulated roots.** Representative confocal images of mutant and WT primary root tips showing *DR5rev:GFP* expression under **(A)** vertical (non-gravistimulated condition), **(B)** 5 h gravistimulation, **(C)** 8 h gravistimulation, and **(D)** 12 h gravistimulation. Yellow arrowheads indicate auxin reporter expression expanding from columella to epidermal cells of the elongation zone. Scale bar = 50 μm. **(E)** Quantification of *DR5rev*:GFP asymmetry as a ratio of fluorescence intensity on the lower and upper flanks of gravistimulated roots at the indicated time points. Values are means from analysis of 10 roots for each time point and error bars indicate standard errors. Similar letters indicate no statistical difference while different letters indicate a statistically significant difference (*P* < 0.05) by Student’s *t*-test.

To test this hypothesis, vertical plate-grown WT and *tno1* seedlings expressing *DR5rev:GFP* were gravistimulated by rotating the plates by 90°. Confocal images were acquired before gravistimulation and subsequently at 5, 8, and 12 h after gravistimulation for both WT and *tno1* root tips (**Figures 5A–D**). In the WT background, GFP fluorescence progressed shootward from the columella toward the lateral rootcap and epidermal cells of the lower side of the root. Conversely, the shootward appearance of fluorescence in *tno1* was severely inhibited and failed to reach WT levels (arrowheads in **Figures 5B–D**). The difference in GFP fluorescence intensity on the upper and lower flanks was expressed as a ratio for each genotype at different time points. This ratio was significantly higher for WT roots than for *tno1* mutants at all time-points, suggesting a stronger response in WT than in the mutants (**Figure 5E**). This in turn implies that the defect in gravitropic bending in *tno1* mutants may be due to a decrease in auxin transport, or a difference in auxin response, from the columella to the lateral rootcap and epidermal cells on the lower side of the gravistimulated root. Such a defect could explain the reduced bending observed in *tno1* roots. Since auxin asymmetry within LR tips has been suggested to occur by a similar mechanism (Rosquete et al., 2013), this could also explain the lower percentage of stage II LRs displaying auxin response asymmetry in *tno1*.

### TNO1 is not Required for Bulk Endocytosis

**FIGURE 6:**
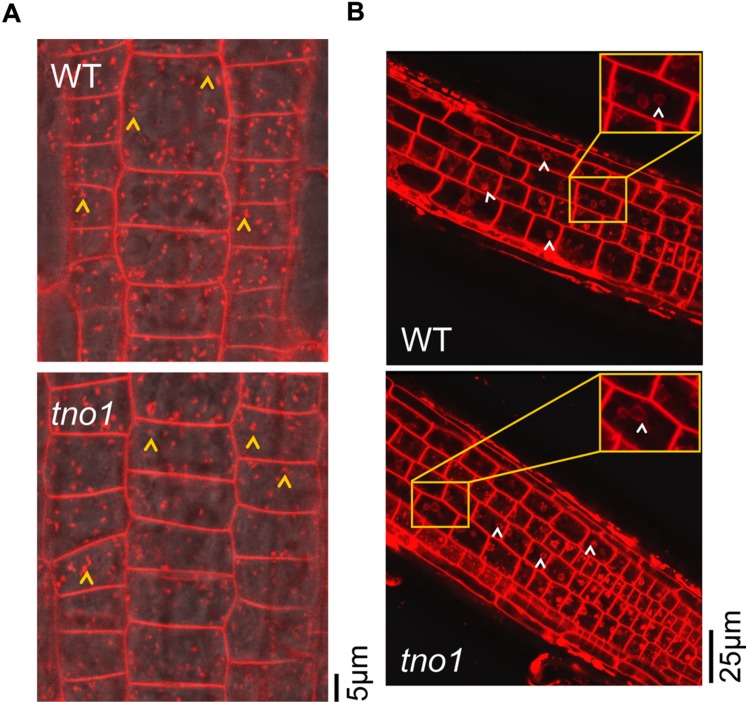
***tno1* mutants have normal bulk endocytosis and arrival of membrane cargo from plasma membrane to Brefeldin A (BFA) bodies.** **(A)** Root cells showing uptake of FM4-64 after 2 min of treatment of 4-day-old seedlings with 4 μM FM4-64 in liquid 0.5× MS medium. Yellow arrowheads indicate early endosomes/TGN. **(B)** Root cells treated with 35 μM BFA for 1 h, followed by a 10 min incubation with 4 μM FM4-64, showing arrival of FM4-64 at BFA bodies. White arrowheads indicate BFA bodies.

Auxin transporters such as the PIN proteins are continuously endocytosed to the TGN and recycled back to the plasma membrane to maintain an appropriate density, thus allowing steady auxin flux through plant cells (Kleine-Vehn and Friml, 2008; Friml, 2010). Defects in endocytosis and components involved in the endocytic route to the TGN can, therefore, cause a defect in auxin transport pathways. Since *tno1* mutants show defects in TGN dynamics (Kim and Bassham, 2011) and display auxin-related defects, we hypothesized that loss of TNO1 might alter endocytic routes and/or arrival of cargo at the TGN. This in turn would manifest in altered auxin transporter trafficking dynamics, and subsequently alter auxin responses. To test this hypothesis, we analyzed endocytosis in root epidermal cells using the lipophilic styryl dye FM4-64, which is endocytosed and labels early endosomes. To capture early endocytic events, roots of 4-day-old seedlings were stained with 4 μM FM4-64 for 2 min and then washed twice for 30 s each time in MS medium. Both WT and *tno1* root cells showed similar endosome labeling patterns (**Figure 6A**, yellow arrowheads), suggesting that bulk endocytic uptake is normal in *tno1* roots.

To confirm that bulk endocytosis was unaffected and that endocytic vesicles are delivered normally to the TGN in the mutant, the incorporation of FM4-64 into BFA bodies was assessed. The fungal toxin BFA causes aggregation of the TGN and TGN-derived endosomes to form BFA bodies (Geldner et al., 2001; Dettmer et al., 2006; Lam et al., 2009). BFA body formation is delayed in *tno1* cotyledons but occurs at similar rates in root cells of WT and *tno1* mutants (Kim and Bassham, 2011). It was hypothesized that if *tno1* has defects in the delivery of endocytosed material to the TGN, then FM4-64 labeling of BFA bodies in *tno1* roots would be reduced compared with WT roots. To test this hypothesis, 5-day-old WT and mutant seedlings were incubated with 35 μM BFA for an hour to allow formation of BFA bodies. They were then stained with 4 μM FM4-64 for 10 min in the presence of BFA, followed by two washes in MS medium containing BFA. The BFA bodies in both WT and *tno1* roots showed normal FM4-64 staining (**Figure 6B**, white arrowheads) after 10 min. Thus, loss of TNO1 does not affect bulk endocytosis and membrane flow from the plasma membrane to the TGN in root cells.

## Discussion

Auxin flow in plants is an important determinant of root architecture and tropic bending such as gravitropism. The polar transport of auxin depends on the specific localization of auxin transporters, which in turn is dependent on the cellular trafficking machinery. The TGN is an important organelle controlling vesicle trafficking, with the presence of multiple proteins that aid the trafficking of cargoes such as auxin transporters. In this study we have demonstrated a role for a putative tethering factor localized at the TGN in efficient gravitropic bending and LR emergence, possibly by affecting auxin transport.

### TNO1 may Regulate Gravitropic Responses through Trafficking of Auxin Transporters

Auxin flows in a polar manner from the shoot to the root tip, via the vasculature, and then back through the cortical and epidermal cell layers shootward. This polar transport is dependent on the polar localization of auxin transporters to plasma membranes, via trafficking pathways that achieve correct subcellular distribution (Kleine-Vehn and Friml, 2008). Auxin transporters undergo endocytic recycling from the plasma membrane as well as being targeted to new plasma membrane domains via the TGN (Kleine-Vehn and Friml, 2008). This enables a plant to respond to environmental cues and adjust its growth pattern accordingly. For example, during the bending of roots upon gravistimulation, an accumulation of auxin in the epidermal layer of the elongation zone in the lower half of the root is required to facilitate the bending process (Tanaka et al., 2006; Peer et al., 2011). This occurs by the relocalization of auxin transporters, AUX1 in the columella and lateral rootcap and AUX1 and PIN2 in the epidermal cells (Strohm et al., 2012). The resulting readjustment of auxin flow from the columella via the lateral rootcap cell to the epidermal cells in the root elongation zone leads to differential growth (Ottenschlager et al., 2003). Since *tno1* has defects in intracellular trafficking and TGN dynamics (Kim and Bassham, 2011), we hypothesize that these may hinder auxin transport pathways due to change(s) in trafficking of auxin transporter(s).

Phenotypic characterization of gravitropic responses in *tno1* roots and hypocotyls showed that these mutants have a decreased angle of bending upon gravistimulation. Pharmacological studies with auxin transport inhibitors and exogenous auxin analogs suggest that altered auxin pathways are likely to be the underlying cause of this defect. The gravitropic bending defect in *tno1* roots and hypocotyls can be rescued by exogenous application of 1-NAA and IAA, perhaps by overriding endogenous auxin levels and routes of polar auxin transport, thus restoring auxin responses. Growth of *tno1* roots is hypersensitive to 2,4-D and IAA but not to the eﬄux-specific substrate 1-NAA or the eﬄux blocker NPA, raising the possibility that the auxin influx pathway might be defective while auxin eﬄux is normal. Although blocking auxin influx with 1-NOA does not result in large differences in root elongation between the *tno1* mutant and WT, the sensitivity to 2,4-D in the presence of 1-NOA is still higher for the *tno1* mutant roots than the WT and complemented lines. This, coupled with the inability of 2,4-D to rescue the gravitropic bending defect of mutant hypocotyls, may suggest altered responses to the influx-specific substrate 2,4-D. Additionally, the delayed progression of *DR5rev:GFP* expression in epidermal cells of gravistimulated *tno1* roots compared to WT roots suggests that auxin flow from the columella to the epidermis (via the lateral rootcap cells) may be reduced in *tno1* roots. Based on these results, we hypothesize that altered auxin influx capacity underlies the gravitropic defects in *tno1*. Given the role of TNO1, this could occur through altered trafficking of auxin influx carrier(s). However, it is also possible that the sensitivity to 2,4-D in the *tno1* mutant is due to differences in auxin signaling, feedback or response, since it is known that the auxin sensing mechanism is complex and varies for different kinds of auxins (Calderón Villalobos et al., 2012).

Since auxin transporters are trafficked via endocytosis (Kleine-Vehn and Friml, 2008), we hypothesized that TNO1 may affect endocytosis from the plasma membrane to the TGN/early endosomes and hence alter transporter trafficking dynamics. Bulk uptake and arrival of a general endocytosis marker at the TGN was unaffected in the *tno1* mutant and thus the auxin-related defects do not appear to be due to defects in bulk endocytosis. However, it is possible that TNO1 is required for the polar targeting of a particular subset of auxin transporter(s), either as they are newly synthesized or during the recycling process for membrane relocalization. Further detailed characterization of the behavior of such proteins in *tno1* cells will help distinguish between these hypotheses.

The polar sorting of auxin transporters in plant cells is highly complex and our understanding of how the SNARE machinery might regulate this polarity is incomplete. Vacuolar SNAREs have been shown to play a role in maintaining auxin maxima and localization of the auxin eﬄux carrier PIN1 in the leaf primordium, thereby regulating leaf vasculature (Shirakawa et al., 2009). The SYP4 family of Qa SNAREs affects auxin distribution, root gravitropism, and the intracellular trafficking of the PIN2 transporter to the vacuole for degradation (Uemura et al., 2012). Since TNO1 associates with the SNARE machinery at the TGN, and its loss causes a mis-localization of SYP61 as well as vacuolar sorting defects (Kim and Bassham, 2011), the SYP41/SYP61/VTI12 complex in conjunction with TNO1 may regulate auxin transport by affecting auxin transporter trafficking and turnover.

### TNO1 Contributes to the Temporal Control of LR Emergence

We have demonstrated that TNO1 is involved in LR emergence and generation of auxin response asymmetry in an emerging LR. LR development begins by division of LR founder cells (single or pairs of pericycle cells facing the xylem poles) in the primary root and subsequent formation of an auxin maximum and increased auxin responsiveness of the founder cells (De Smet et al., 2007; Dubrovsky et al., 2008; Peret et al., 2009). Auxin transport plays a crucial role in LR emergence, with both auxin influx and eﬄux carriers being involved. The auxin influx carrier AUX1 is important for loading auxin into the vascular system (Marchant et al., 2002) and *aux1* mutants have an almost 50% reduction in LR number. Auxin in the endodermis and cortical cells induces another auxin influx carrier, LAX3, which also facilitates LR emergence (Swarup et al., 2008). The concerted action of AUX1 and LAX3, with subsequent induction of expression of PIN3 in the cortex, leads to emergence of the LR from the primary root (Swarup and Peret, 2012; Peret et al., 2013).

The LR emergence defects observed in *tno1* are consistent with altered auxin transport due to disruption in the trafficking or targeting of auxin transporters. *tno1* mutants have a significantly reduced percentage (40% vs. 80% in WT) of emergent Stage II LRs with asymmetric *DR5rev:GFP* expression, raising the possibility of auxin transport defect(s). Since the action of PIN transporters plays a crucial role in establishing this asymmetric pattern of auxin activity in emergent LRs (Rosquete et al., 2013), TNO1 might affect the trafficking of PIN transporters in the LR tip cells during this process.

LR emergence occurs within the pericycle and involves degradation of the pectin-rich middle lamella, with subsequent cell separation to allow growth of the LR through multiple cell layers (Vilches-Barro and Maizel, 2014). Focused auxin flow via transporters into the cortex and the epidermis causes an induction of expression of cell wall remodeling enzymes (Swarup et al., 2008; Peret et al., 2013). Secretory cargo, including various cell wall remodeling enzymes, traffics via the Golgi and TGN toward the extracellular space (Foresti and Denecke, 2008; Toyooka et al., 2009; De Caroli et al., 2011), in a pathway potentially mediated by SYP61 (Drakakaki et al., 2012). Hence, it is possible that defects in the TGN, including the mis-localization of SYP61 seen in *tno1*, could also lead to disrupted secretion of cell wall remodeling enzymes, thus leading to slower LR emergence (Worden et al., 2012).

The identification of effectors directly associated with TNO1 may help elucidate its mechanism of action during these auxin-mediated processes. Since tethering factors can interact with Rabs (Markgraf et al., 2007), TNO1 could potentially indirectly affect auxin transporter sorting by affecting the Rab cycle or the function of Rab effectors. Selective targeting of auxin transporters between the plasma membrane and the TGN could be defective in *tno1* independent of the constitutive pathways that constantly recycle them between these membranes. Turnover of auxin transporters could also be reduced due to defective targeting to the vacuole for degradation. Analysis of the trafficking of specific auxin transporters in the *tno1* mutant would help to distinguish between these possibilities.

## Author Contributions

RR and DB designed the experiments. RR collected and analyzed the data. RR and DB interpreted the data and wrote the manuscript.

## Conflict of Interest Statement

The authors declare that the research was conducted in the absence of any commercial or financial relationships that could be construed as a potential conflict of interest.
